# Impact of Ostiomeatal Complex Anatomical Variants on Histological Remodeling and Radiological Severity: A Retrospective Cross-Sectional Study

**DOI:** 10.7759/cureus.111255

**Published:** 2026-06-21

**Authors:** Mohammed A Mohammed

**Affiliations:** 1 Human Anatomy, Hammurabi College of Medicine, University of Babylon, Babylon, IRQ

**Keywords:** anatomical variations, chronic rhinosinusitis, computed tomography, concha bullosa, eosinophilic inflammation, functional endoscopic sinus surgery, lund-mackay score, mucosal remodeling, ostiomeatal complex, subepithelial basement membrane

## Abstract

Background

The anatomical integrity of the ostiomeatal complex (OMC) is critical in the pathophysiology of chronic rhinosinusitis (CRS). While individual structural deformities are known to impede sinus drainage, the cumulative "dose-response" impact of concurrent OMC variants on microscopic mucosal remodeling remains under-investigated.

Objective

To quantitatively map the correlation between specific OMC anatomical variants - nasal septal deviation (NSD), concha bullosa (CB), and agger nasi (AN) cells - and the severity of histological tissue remodeling and radiological opacification.

Methods

This retrospective cross-sectional study evaluated archived medical, radiological, and histopathological records of 80 adult patients (January 2024 to February 2026). The cohort comprised an experimental group (n=60) with CT-confirmed OMC variants and a normal control group (n=20). To isolate anatomical effects, patients with a history of severe atopy or heavy smoking were excluded. Data extracted included the Lund-Mackay score, subepithelial basement membrane (SBM) thickness, eosinophil count/high-power field (HPF), and goblet cell density/HPF. Statistical evaluation utilized one-way analysis of variance (ANOVA) to determine the dose-response effect of single versus multiple anatomical variants.

Results

All individual OMC variants significantly exacerbated pathological markers compared to controls (p<0.001). A highly significant linear dose-response relationship (p for trend <0.001) demonstrated that mucosal degradation worsened with increasing structural complexity. In the triple variant group (n=24), where NSD, CB, and AN coexisted, radiological and histopathological severity peaked significantly compared to controls (n=20): the Lund-Mackay score advanced to 14.6 ± 2.5 (vs. 3.6 ± 1.1), SBM thickness increased to 15.3 ± 1.8 μm (vs. 10.1 ± 1.0 μm), mucosal eosinophil influx escalated to 9.8 ± 2.2 cells/HPF (vs. 2.4 ± 0.8 cells/HPF), and goblet cell density expanded to 34.2 ± 5.0 cells/HPF (vs. 21.3 ± 3.4 cells/HPF). Transitioning from a single variant (n=19) to the full triad significantly accelerated tissue fibrosis and inflammatory infiltration (p<0.01).

Conclusions

Anatomical complexity within the OMC acts as a direct, cumulative driver of chronic sinus pathology and severe mucosal remodeling. The linear escalation of tissue degradation highlights the clinical necessity of early surgical or medical intervention in patients presenting with multiple concurrent variants to prevent irreversible structural damage.

## Introduction

Chronic rhinosinusitis (CRS) remains one of the most prevalent debilitating conditions in otorhinolaryngology, characterized by persistent mucosal inflammation and significant impairment of patient quality of life [1]. At the heart of CRS pathophysiology lies the ostiomeatal complex (OMC), a functional entity of the anterior ethmoid region that serves as the final common pathway for drainage and ventilation of the frontal, maxillary, and anterior ethmoid sinuses [2]. The "OMC theory" posits that any structural compromise within this narrow anatomical bottleneck can lead to a vicious cycle of impaired mucociliary clearance, stasis of secretions, and chronic inflammatory changes in the dependent sinus mucosa [3]. While the role of mechanical obstruction is well-established, the influence of specific anatomical polymorphisms remains a subject of intense clinical debate [4]. Among these, nasal septal deviation (NSD), concha bullosa (CB), and agger nasi (AN) cells are the most frequently encountered variants [5]. These structures do not merely act as physical barriers; they alter the sinonasal microenvironment by inducing turbulent airflow and localized pressure changes, which may serve as a mechanical trigger for chronic mucosal stress [6]. However, the extent to which these variations independently or cumulatively contribute to tissue-level damage is not yet fully elucidated.

In the realm of modern rhinology, the concept of "mucosal remodeling" has emerged as a critical marker of disease severity [7]. This process involves profound structural alterations, including the thickening of the subepithelial basement membrane (SBM), hyperplasia of the Goblet cells, and intense infiltration of inflammatory cells, particularly eosinophils [8]. These histological markers are not merely passive features of inflammation but are indicators of a profound shift in the mucosal architecture that can influence the success of surgical interventions like functional endoscopic sinus surgery (FESS) [9]. Despite numerous radiological studies, there is a relative scarcity of research that quantifies the "anatomical burden" the cumulative impact of multiple concurrent variants on these specific histopathological parameters. From a human anatomy perspective, understanding whether a "dose-response" relationship exists between anatomical complexity and mucosal deterioration is essential for personalizing surgical approaches and predicting postoperative outcomes [10].

Therefore, the primary objective of this retrospective study is to evaluate the association between specific OMC anatomical variants (NSD, CB, and AN cells) and the severity of histological and radiological changes in patients with CRS. We hypothesize that there is a possible dose-response relationship, where an increasing number of concurrent anatomical variations correlates with more severe chronic mucosal remodeling. Importantly, given the retrospective nature of this study, our aim is to explore these clinical and histopathological associations rather than to establish direct causality.

## Materials and methods

Study design and setting

This is a retrospective cross-sectional study conducted at the Department of Human Anatomy, Hammurabi College of Medicine, University of Babylon, Iraq. The research involved the quantitative extraction and anatomical analysis of archived medical, radiological, and histopathological records. The records spanned a period from January 2024 to February 2026, with the final data extraction and analysis concluding three months prior to the drafting of this manuscript. The primary objective was to evaluate the correlation between anatomical variants of the OMC and quantitative mucosal remodeling.

Ethical considerations

The study protocol was reviewed and formally approved by the Institutional Ethics Committee of the Hammurabi College of Medicine (approval No: 63 in 11/5/2026). Given the retrospective nature of the study, patient data were fully anonymized to ensure privacy, and the research was conducted in strict adherence to the ethical guidelines of the Declaration of Helsinki.

Data source and patient selection

Records for a total of 80 patients were retrieved from authenticated hospital databases. To ensure high clinical reliability, the data belonged to patients who had been previously evaluated, diagnosed, and surgically managed by a standard multidisciplinary medical team (comprising ENT surgeons, radiologists, and pathologists).

**Figure 1:**
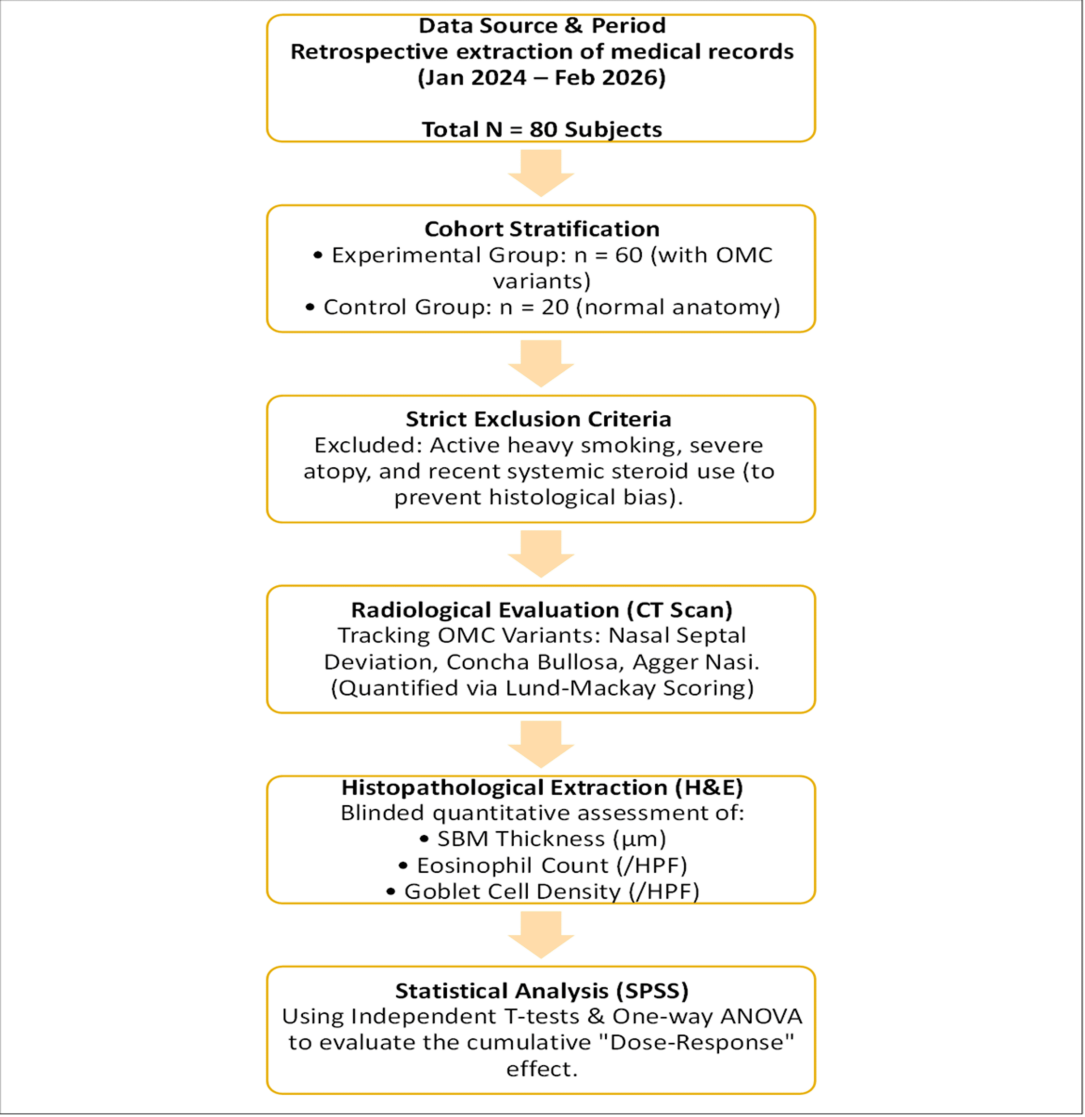
Flowchart of the study methodology Flowchart detailing the comprehensive workflow of the study, including the retrospective retrieval of 80 patient records from hospital databases, stratification into the experimental group (n=60) and control group (n=20), and the rigorous application of clinical, radiological, and histopathological inclusion and exclusion criteria. OMC: ostiomeatal complex. Image credit: Image created by the author using Microsoft PowerPoint (Microsoft Corp., Redmond, WA, USA).

The data extraction workflow, patient selection process, exclusion criteria, and cohort stratification are comprehensively illustrated in the study methodology flowchart (Figure 1).

Based on this structured workflow, the extracted records were stratified into two distinct clinical groups. The experimental group (n=60) comprised records of patients diagnosed with CRS who underwent FESS and presented with CT-confirmed anatomical variants of the OMC, including NSD, CB, or AN. Conversely, the control group (n=20) consisted of records of individuals who underwent nasal procedures for non-inflammatory and non-sinus conditions, such as cosmetic rhinoplasty or functional septoplasty for trauma, allowing for the incidental sampling of middle meatal/turbinate mucosa during standard surgical access. These patients demonstrated no clinical or radiological evidence of OMC obstruction or mucosal disease and lacked any preoperative CRS-associated symptoms.

To accurately isolate the independent impact of OMC anatomical variations on mucosal tissue and to minimize the effect of the confounding variables, strict eligibility criteria were applied. Inclusion required adult patients over 18 years of age with complete and matching radiological CT and histopathological documentation. Patients were strictly excluded if their records indicated a history of previous sinonasal surgery, cystic fibrosis, sinonasal tumors, or systemic steroid use within the four weeks preceding their surgery. Furthermore, to prevent histological bias, patients with a documented history of active heavy smoking, severe systemic asthma, or severe atopy (allergic rhinitis) were excluded. This rigorous selection process ensured that the observed eosinophilia and SBM thickening were primarily driven by anatomical obstruction rather than systemic or environmental factors.

Radiological and anatomical evaluation

The anatomical mapping of each case was based on high-resolution CT reports provided by the hospital's senior radiologists. The researcher tracked the presence of specific anatomical variants (NSD, CB, and AN) and the overall severity of sinus opacification, which was quantified using the standard Lund-Mackay scoring system [11].

Histopathological data extraction and blinding

Validated quantitative metrics were extracted from official pathology laboratory reports. The original tissue biopsies were harvested by the surgical team from the middle meatus area and processed using standard Hematoxylin and Eosin (H&E) staining. Crucially, the original histopathological evaluations were performed by the hospital's pathologists for routine diagnostic purposes, completely blinded to the specific anatomical objectives of the current study. This eliminated any observer bias in the quantification of: (1) SBM thickness: measured in micrometers (μm) using standard institutional digital microscopic analysis across multiple fields; (2) Eosinophil count: quantified manually per high-power field (HPF) by the independent pathologists; (3) Goblet cell density: assessed manually per HPF to ensure inter-observer reliability within the institutional protocol.

Statistical analysis

Statistical analysis of the extracted data was performed using IBM SPSS Statistics for Windows, version 26.0 (Released 2019; IBM Corp., Armonk, New York, United States). Continuous variables were expressed as mean ± standard deviation (SD). Differences between groups were analyzed using independent t-tests and one-way ANOVA to evaluate both the independent and cumulative (dose-response) effects of the anatomical variants. A p-value of <0.05 was considered statistically significant.

## Results

Radiological and histopathological profile of individual OMC variants

This section evaluates the independent impact of each anatomical variant compared to the control group. The data identify the specific pathological burden associated with NSD, CB, and AN cells. Comparative analysis revealed that all individual abnormal structural cohorts exhibited a statistically significant inflation in tissue remodeling parameters relative to healthy controls (p<0.001). Among the individual variants, patients with CB demonstrated the highest radiological and histopathological severity, followed closely by the AN and NSD cohorts, demonstrating that every structural anomaly acts as an active contributor to mucosal pathology (Table 1).

**Table 1:** Radiological and histopathological profile of individual variants Data are expressed as mean ± SD. All individual variant groups demonstrated highly significant differences compared to the control group (p<0.001).

Clinical / Histological marker	Control group (n=20)	Nasal septal deviation group (n=59)	Concha bullosa (n=43)	Agger nasi (n=35)
Lund-Mackay score	3.6 ± 1.1	11.8 ± 3.2	13.9 ± 2.8	12.9 ± 3.0
SBM thickness (µm)	10.1 ± 1.0	13.8 ± 1.6	15.0 ± 1.4	14.3 ± 1.5
Eosinophil count (/HPF)	2.4 ± 0.8	7.1 ± 2.0	9.0 ± 1.9	8.1 ± 2.1
Goblet cell density (/HPF)	21.3 ± 3.4	30.5 ± 5.1	33.4 ± 4.5	32.0 ± 4.8

Pathological severity in patients with concurrent triple variants

**Figure 2:**
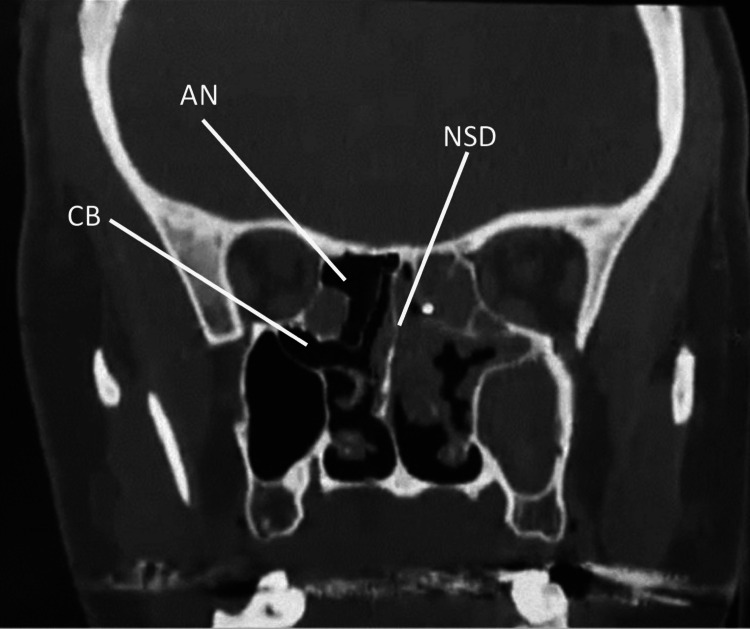
Coronal computed tomography (CT) scan of the paranasal sinuses demonstrating a cumulative anatomical variant burden within the ostiomeatal complex (OMC) White arrows indicate a prominent concha bullosa (CB), an agger nasi (AN) cell, and significant nasal septal deviation (NSD) to the opposite side, resulting in mechanical bottleneck obstruction and subsequent sinus opacification.

To determine the maximum impact of anatomical obstruction, patients presenting with all three variants simultaneously (triple variant group, n=24) were compared directly against the anatomically normal control group (n=20). The coexistence of the full triad (NSD + CB + AN) within the OMC culminated in profound tissue degradation. The Lund-Mackay score peaked at an advanced mean of 14.6 ± 2.5 compared to 3.6 ± 1.1 in controls (p<0.001). This radiological opacification was mirrored by severe structural alterations, including a highly accelerated SBM thickness of 15.3± 1.8 μm and a massive inflammatory recruitment of mucosal eosinophils (9.8 ± 2.2 cells/HPF), confirming that the synergistic impact of a total mechanical blockade culminates in advanced mucosal remodeling. The complex radiological architecture of these coexisting variants is illustrated in Figure 2.

The associated quantitative histopathological parameters are outlined in Table 2.

**Table 2:** Pathological severity in the triple variants The triple cariant group represents the most severe anatomical obstruction (nasal septal deviation + concha bullosa + agger nasi). Statistical significance was maintained across all radiological and histological parameters.

Clinical / Histological marker	Control group (n=20)	Triple variant group (n=24)	p-value
Lund-Mackay score	3.6 ± 1.1	14.6 ± 2.5	<0.001
Subepithelial basement membrane (SBM) thickness (µm)	10.1 ± 1.0	15.3 ± 1.8	<0.001
Eosinophil count (/HPF)	2.4 ± 0.8	9.8 ± 2.2	<0.001
Goblet cell density (/HPF)	21.3 ± 3.4	34.2 ± 5.0	<0.001

Comparative analysis: single vs. triple anatomical variants

This comparison highlights the progression of tissue damage as anatomical complexity increases, contrasting patients with only one variant (n=19) against those with the full triad of obstructions (n=24). The transition from a single structural anomaly to a triple variant pattern resulted in a sharp, significant escalation across all markers (p<0.01). The total radiological sinus burden increased by a mean difference of +5.8 points on the Lund-Mackay scale. Similarly, the hyperplastic change in the goblet cells demonstrated a substantial mean difference of +9.1 cells/HPF in the triple variant group, proving that a higher structural variant density directly intensifies cellular remodeling, with clear variations captured in Table 3.

**Table 3:** Single vs. triple anatomical variants Increasing the number of variants from one to three significantly exacerbates subepithelial fibrosis and eosinophilic inflammation (p<0.01).

Clinical / Histological marker	Single variant (n=19)	Triple variant (n=24)	Mean difference
Lund-Mackay score	8.8 ± 2.1	14.6 ± 2.5	5.8
Subepithelial basement membrane (SBM) thickness (µm)	11.8 ± 1.2	15.3 ± 1.8	+3.5
Eosinophil count (/HPF)	4.4 ± 1.2	9.8 ± 2.2	5.4
Goblet cell density (/HPF)	25.1 ± 3.8	34.2 ± 5.0	9.1

Dose-response relationship of anatomical complexity

The following master table establishes a linear trend, showing that each additional anatomical variant in the OMC area contributes to a progressive and significant deterioration of the nasal mucosa (Table 4).

**Table 4:** Dose-response relationship of anatomical complexity A highly significant linear trend (p<0.001) confirms that anatomical complexity is a direct driver of pathological mucosal remodeling.

Marker	Control (zero variants; n=20)	Single (one variant; n=19)	Double (two variants; n=17)	Triple (three variants; n=24)	p-value (Trend)
Lund-Mackay Score	3.6 ± 1.1	8.8 ± 2.1	11.5 ± 2.2	14.6 ± 2.5	<0.001
Subepithelial basement membrane (SBM) thickness (µm)	10.1± 1.0	11.8 ± 1.2	13.8 ± 1.3	15.3 ± 1.8	<0.001
Eosinophil count	2.4 ± 0.8	4.4 ± 1.2	6.7 ± 2.3	9.8 ± 2.2	<0.001
Goblet cell density	21.3± 3.4	25.1 ± 3.8	30.4 ± 3.2	34.2 ± 5.0	<0.001

Statistical evaluation via trend analysis confirmed an uncompromised, stepwise progression from healthy controls (zero variants) up to the triad cohort (three variants; p trend<0.001). Every progressive structural layer linearly forced an expansion in SBM thickness (10.1 ± 1.0 μm ➔ 11.8 ± 1.2 μm ➔ 13.8 ± 1.3 μm ➔ 15.3 ± 1.8 μm) and synchronized seamlessly with an escalating cellular influx of tissue eosinophils, validating the cumulative dose-response nature of anatomical blockages, which exhibits a highly significant linear trend.

## Discussion

The anatomical integrity of the OMC is the pathophysiological cornerstone of sinonasal health. The primary objective of this retrospective cross-sectional study was to evaluate the association between specific OMC anatomical variants - NSD, CB, and AN - and the severity of histological remodeling and radiological opacification. Our findings align with the mechanical obstruction theory in CRS and highlight a possible "dose-response" relationship between the cumulative anatomical burden and the degree of microscopic mucosal deterioration.

When evaluating the variants independently, our data revealed that all three structures are significantly associated with exacerbated mucosal disease compared to the normal anatomical control group. Interestingly, CB exhibited the most profound independent association with SBM thickness (15.0 ± 1.4 µm) and eosinophilic infiltration (9.0 ± 1.9/HPF). This aligns with the aerodynamic theory of the nasal cavity, which suggests that a pneumatized middle turbinate creates a direct, expansive mechanical compression on the uncinate process and the infundibulum [12]. Unlike NSD, which primarily alters laminar airflow, the volumetric expansion of a CB severely compromises the microenvironment of the middle meatus, potentially leading to localized hypoxia and impaired mucociliary clearance, which are well-documented triggers for tissue-level inflammation [13].

The most compelling finding of our research is the strong linear correlation between the number of concurrent anatomical variants and the progression of disease severity. Patients harboring the "triple variant" (NSD + CB + AN) demonstrated extreme pathological parameters, including a Lund-Mackay score of 14.6 ± 2.5, SBM thickness of 15.3 ± 1.8 µm, and an eosinophil count of 9.8 ± 2.2/HPF. The progression from a single variant to a triple variant yielded a highly significant increase across all measured metrics (p<0.001). From an anatomical and histopathological standpoint, this supports the "multi-hit hypothesis" in sinonasal disease. A single variant may partially obstruct drainage, allowing the mucosa to compensate. However, the superimposition of multiple variants may act synergistically to critically narrow the OMC bottleneck [14]. This stasis traps environmental antigens and pathogens, potentially initiating a chronic inflammatory cascade characterized by continuous eosinophilic degranulation and subsequent fibroblast activation, which is associated with SBM thickening [15].

In this study, goblet cell hyperplasia and SBM thickening were utilized as quantitative markers of chronic mucosal remodeling. Goblet cell density increased drastically in the triple variant group (34.2 ± 5.0/HPF) compared to controls (21.3 ± 3.4/HPF). Goblet cell hyperplasia is an adaptive structural response to turbulent airflow and chronic irritation, designed to hyper-secrete mucin to trap foreign particles [16]. However, in a physically obstructed OMC, this hyper-secrete becomes pathological, contributing to mucus stasis. Furthermore, the significant SBM thickening observed in our experimental groups reflects a transition from acute, reversible inflammation to chronic fibrotic remodeling [17]. Recognizing this microscopic damage is clinically paramount, as it indicates that highly obstructed patients may require more aggressive postoperative medical management (e.g., topical corticosteroids) to manage cellular-level inflammation even after the macroscopic anatomical defects are corrected via FESS [18].

While our findings provide robust quantitative insights, certain limitations must be acknowledged. The retrospective, cross-sectional design restricts our ability to establish absolute causality between anatomical variations and longitudinal disease progression. Furthermore, the retrospective nature of this study presents additional significant limitations, including the unequal numbers in the study and control groups, the exclusion of the comprehensive clinical presentation of both patients and controls, and the selection of 'normal' control participants based solely on radiologically normal CT scans with no explicit mention of whether these patients had any CRS-associated symptoms. Additionally, the opportunistic sampling of sinus mucosa from the control group for histopathological examination lacked uniform standardization. Another notable limitation is the absence of original matched histological images for the patients included in our dataset. Due to the hospital's strict archiving policy, histological tissue slides are routinely discarded after a maximum period of one month. Conversely, the radiological figures (CT scans) included in this study represent authentic clinical examples from our exact patient cohort, perfectly matched with their statistical data.

## Conclusions

In conclusion, this study provides quantitative anatomical and histopathological evidence highlighting a significant association between anatomical variations within the OMC and chronic mucosal remodeling. Considering the limitations of this retrospective study design and its sequelae, including the choice and number in the control group and not taking into account the clinical features among study patients and controls, we can only conclude that there is a possibility of a "dose-response relationship" from single to triple anatomical OMC variations as the drivers of chronic mucosal remodeling. From a clinical perspective, these findings suggest that identifying a high anatomical burden in patients could alert the surgeon to the presence of underlying mucosal fibrosis, potentially necessitating a comprehensive surgical approach and postoperative anti-inflammatory therapy. However, there is a clear need for further multicentric prospective studies to confirm this hypothesis.
